# Knowledge mapping of the research on lexical inferencing: A bibliometric analysis

**DOI:** 10.3389/fpsyg.2023.1101241

**Published:** 2023-01-18

**Authors:** Hui Yang, Lin Fan, Hongshan Yin

**Affiliations:** ^1^School of Foreign Languages, Qingdao University of Science and Technology, Qingdao, China; ^2^Artificial Intelligence and Human Languages Lab, Beijing Foreign Studies University, Beijing, China

**Keywords:** lexical inferencing, bibliometric analysis, knowledge domain, intellectual base and structure, hot topics

## Abstract

Lexical inferencing functions as one of the most important and effective skills used in language comprehension pertaining to psychological, cognitive and neurological aspects. Given its complex nature and crucial role in language comprehension, lexical inferencing has received considerable attention. The present study visualized the knowledge domain of the research on lexical inferencing based on a total of 472 articles collected from Web of Science (WoS) Core Collection of Thomson Reuters from 2001 to 2021. The bibliographic data were analyzed through co-cited articles, co-citation clusters of references, and co-occurring keywords to identify holistic intellectual landscape of lexical inferencing with special focus on its intellectual structure and base, and hot research topics. The main intellectual base includes probability of activating lexical inferencing in working memory and encoding in long-term memory, the role of lexical inferencing in reading comprehension, in connected speech, in children’s derivation under pragmatic context, and in psychological and neurocognitive processes underlying language processing mechanism. Hot topics are comprised the impacts of lexical inferencing on language acquisition and comprehension (written and spoken language comprehension), the factors (context variables, vocabulary knowledge, and morphological awareness) affecting the presence and efficacy of lexical inferencing, and the time course of lexical inferencing during reading. Critically, the results of this study demonstrated that the contribution of lexical inferencing to language comprehension was strongly correlated with learner-related and discourse-related variables. The study shed valuable light on the understanding of the intellectual background and the dynamic patterns of lexical inferencing over the past two decades, thereby future work in lexical inferencing is suggested as well.

## 1. Introduction

Oxford described lexical inference (Oxford, 1990, p. 47) as “using a variety of linguistic and nonlinguistic clues to guess the meanings of all the words when the learner does not know them.” Haastrup (1991) defined lexical inferencing as the processes of making informed guesses about the meaning of a word on the basis of all the linguistic cues available and the readers’ general knowledge. Lexical inferencing, in essence, denotes the process of inferring implicit meaning based on the available information. Given its significant contribution to language comprehension, lexical inferencing has become an increasingly important topic and has drawn increasing attention from a number of researchers in diverse fields. Over the past two decades, a growing number of studies have investigated the complex roles of lexical inferencing in language comprehension. No research to date, however, has yet been conducted to provide a macroscopic and quantitative overview of the knowledge domain of lexical inferencing research.

Thus, the purpose of the study is to visualize and analyze the knowledge domain of lexical inferencing through CiteSpace. Specifically, this work sets out to systematically overview the co-cited articles, co-citation clusters of references, and co-occurring keywords in the hope of identifying holistic intellectual landscape of lexical inferencing with special interest in its intellectual structure and base, as well as its hot research topics.

## 2. Literature review

The existing literature, on one hand, has documented the direct and indirect effects of lexical inferencing on written and spoken language comprehension (e.g., Gaskell and Marslen-Wilson, 1996, 1998; Ranbom and Connine, 2007; Prior et al., 2014; Ke and Koda, 2017; Chen, 2018; Zhang and Koda, 2018; Zhang et al., 2021). When readers or interlocutors encountered unfamiliar vocabulary in written or oral forms, they would draw on background knowledge and context information to infer the meaning of the unfamiliar word. The ability to infer the unknown meanings of the words undoubtably facilitates readers’ or interlocutors’ language comprehension process. Further, lexical inferencing ability has been captured to play a mediating role in language comprehension. Zhang and Koda (2012) observed that morphological awareness alone made no direct contribution to the readers’ reading comprehension, whereas its modulating effect through the readers’ lexical inferencing ability and vocabulary knowledge on their reading comprehension was found. Zhang (2015) reported that morphological awareness facilitated the readers’ reading vocabulary acquisition *via* lexical inferencing. However, both significant and insignificant correlation were found between morphological awareness and lexical inferencing in reading comprehension (Chen, 2018). Although the inconsistent results were presented, these studies provided insightful information on disentangling the direct and indirect role of lexical inferencing in language comprehension.

On the other hand, the role of lexical inferencing has been extensively examined across different fields. For instance, some researchers explored how and when the inferencing is generated and retained in working memory and encoded in long-term memory during reading and speaking (e.g., Klin et al., 1999a; Calvo, 2005; Currie and Muijselaar, 2019). The findings showed that lexical inferencing was more likely to be activated on-line and maintained in the long-term memory during reading under certain contexts, such as highly predictable context. Researchers also attempted to account for why children find it difficult to infer lexical meanings in pragmatic contexts (Barner et al., 2011; Bott et al., 2012; Stiller et al., 2015). Barner et al. (2011) concluded that children’s knowledge of scalar implicatures constrained their ability to infer lexical meanings. However, Stiller et al.’s (2015) found that children were able to compute implicatures (lexical items such as “some” and “all”) and claimed that contradictory findings may be attributed to the different constructions and tasks used in prior studies. Gaskell and Marslen-Wilson (1996, 1998) investigated phonology-lexicon interface with special focus on matching process between the perceptual input and a form-based lexical representation. The results suggested that on-line inference was involved in mapping speech onto lexical representation.

Abundant research has been performed to explore the effects of lexical inferencing in language comprehension across a wide range of linguistic and psychological fields. The findings of prior studies revealed that a number of influential factors may affect lexical inferencing process during language comprehension. One of the influential factors is learners’ language proficiency level (e.g., Bengeleil and Paribakht, 2004; Kaivanpanah and Alavi, 2008; Fan et al., 2017; Zhang and Lin, 2021). Chen (2018) investigated the contribution of morphological awareness to lexical inferencing during reading among 73 participants aged between 18 to 22 years. The results showed that differences in L2 learners’ language skills could cause the unbalanced facilitation of morphological awareness to lexical inferencing. Vocabulary knowledge has been documented to be another factor impacting lexical inferencing process (e.g., Nassaji, 2003; Nassaji, 2006; Nassaji, 2007; Prior et al., 2014; Zhang and Pei, 2022). Both depth and breadth of vocabulary knowledge were observed to be positively correlated with lexical inferencing and depth of vocabulary knowledge was found to be a stronger predictor of lexical inferencing success (Marzban and Hadipour, 2012). Nassaji (2006) also found depth of vocabulary knowledge could facilitate lexical inferencing during reading. Learners’ sensitivity to morphological structure affects lexical inferencing as well (e.g., Zhang and Koda, 2012; Zhang, 2015; Ke and Koda, 2017; Chen, 2018, 2019; Zhang et al., 2021, 2022). Effects of first language (L1) lexical knowledge is another key influencing factor for lexical inferencing success (e.g., Paribakht, 2005; Tang and Chan, 2022). Koda and Miller (2018) indicated that readers developed an abstract but sharable awareness of linguistic structure through their cumulative experience in their L1 and exposure to L1 text, which could be utilized in their later-acquired languages. In addition, working memory capacity (e.g., Daneman and Carpenter, 1980; Singer and Ritchot, 1996; Schmalhofer et al., 2002; Cain et al., 2004; Calvo, 2005; Monetta et al., 2008; Currie and Muijselaar, 2019; Kim et al., 2022), contextual constraints (e.g., Gaskell and Marslen-Wilson, 1996, 1998; Tang and Chan, 2022; Zhang et al., 2022), and vocabulary transparency (e.g., Pulido, 2007; Chen, 2019; Chen et al., 2020; Tang and Chan, 2022) are crucial indicators of lexical inferencing success in language comprehension.

Although extensive research has been carried out on lexical inferencing, no single study exists which adequately encompassed the overall profile of lexical inferencing domain. This study aims to construct a bibliometric network and visualization to reveal the scope and structure of lexical inferencing research. Visualizing this scientific domain can highlight the most influential documents, co-citation references, and the most frequently explored topics in terms of co-citation counts, which uncovers potentially significant intellectual structure, dynamic patterns of lexical inferencing. The network analysis of academic literature characterizes the relationship between lexical inferencing and language comprehension, and ascertains the course of the development of lexical inferencing research.

## 3. Methods

### 3.1. Data retrieval

The bibliometric articles were retrieved from Web of Science (WoS) Core Collection of Thomson Reuters, consisting of Science Citation Index Expanded (SCI-EXPANDED), Social Sciences Citation Index (SSCI), Arts and Humanities Citation Index (A&HCI), Conference Proceedings Citation Index - Science (CPCI-S), as well as Conference Proceedings Citation Index - Social Science and Humanities (CPCI-SSH). The WoS database is one of the indexes available and widely used by researchers to access the world’s leading scholarly literature (Falagas et al., 2008; Singh et al., 2021). The dataset was refined by the following search strategies:

Topic = “lexical inferencing,” which searches title, abstract, or keywords.

Time span = “2001–2021.”

Language = “English.”

Document type = “article” or “review article” (excluding “book review”).

The search yields 763 potentially relevant articles distributed in 84 WoS categories. Our major interest was oriented to investigating lexical inferencing in language processing, which led us to remove the articles published in journals specialized in computer science, technology, anthropology, business finance, etc., and selected those published in journals in the fields of linguistics, psychology, neurology, and education. This screening process left us with 545 papers. Finally, after duplicate removal through CiteSpace, a list of 472 articles of topical relevance was used for bibliometric analysis in the domain of lexical inferencing.

### 3.2. Instrument

The visual exploration was performed by means of CiteSpace (v. 6.1.R2), a special-purpose citation analysis tool, to systematically review relevant literature and scientific domain of lexical inferencing. CiteSpace, developed by Chen (2004), can provide a holistic and historical knowledge domain and capture the dynamics of evolving specialties (Chen, 2004, 2017). The current study presented a bibliographic landscape of lexical inferencing research based on co-citation and co-occurrence analysis *via* CiteSpace. A document co-citation map and a keyword co-occurrence network were analyzed to identify the critical references, intellectual base, and hot topics in the domain of lexical inferencing.

## 4. Results

### 4.1. Temporal distribution of bibliometric records and top sources of publications

**Figure 1:**
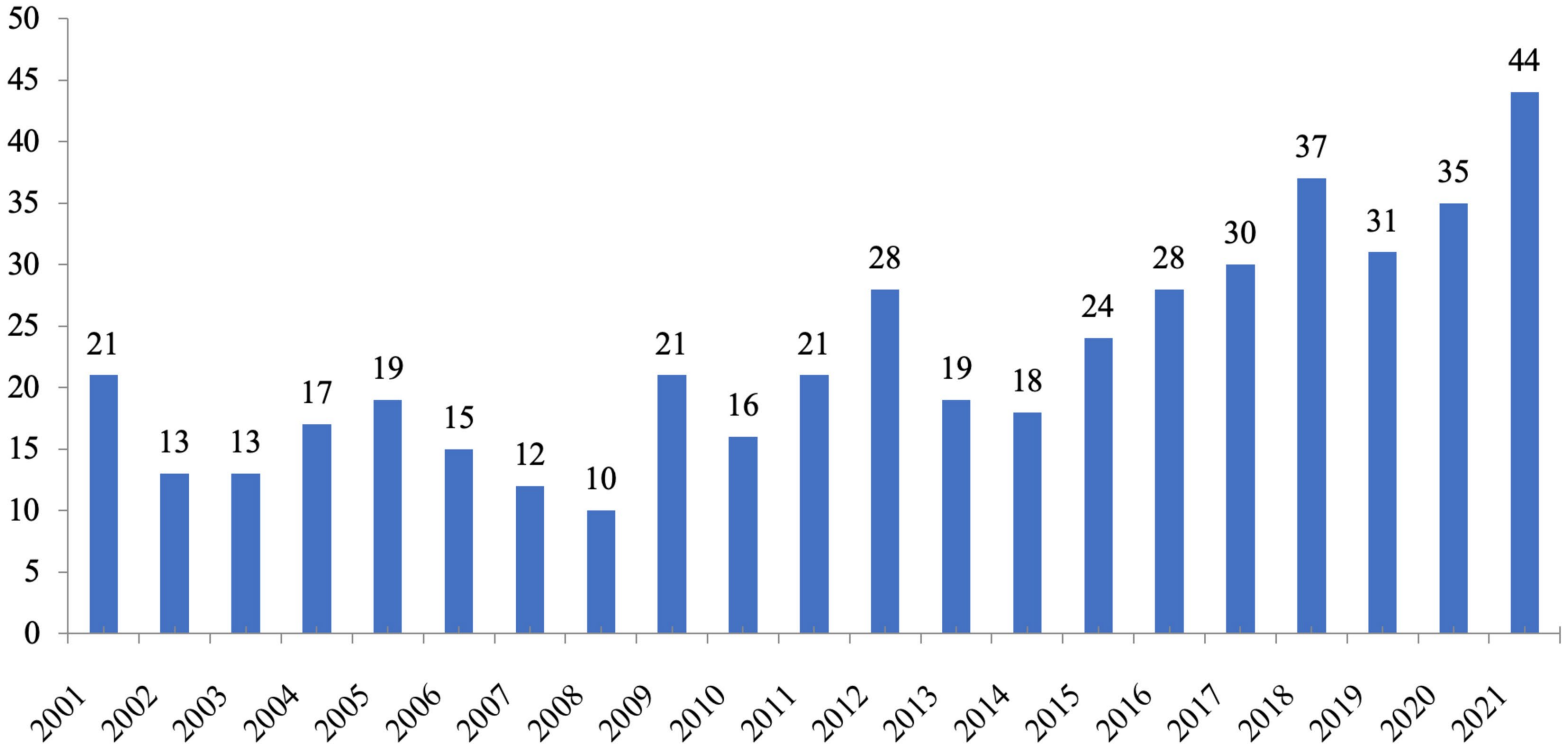
Annual publications regarding lexical inferencing in WoS.

The annual distribution of lexical inferencing over the past two decades, along with the number of published works each year, is illustrated in Figure 1. What can be clearly seen in Figure 1 is the fluctuating growth in annual publications. From 2001 to 2016, the number of annual publications was around 10–28. However, from 2017 onward, there was an increase in annual publications, with 44 articles published in 2021, more than twice the average of those in the duration of 2001–2016. The annual increase of publications suggests the growing interest among researchers in lexical inferencing research.

**Figure 2:**
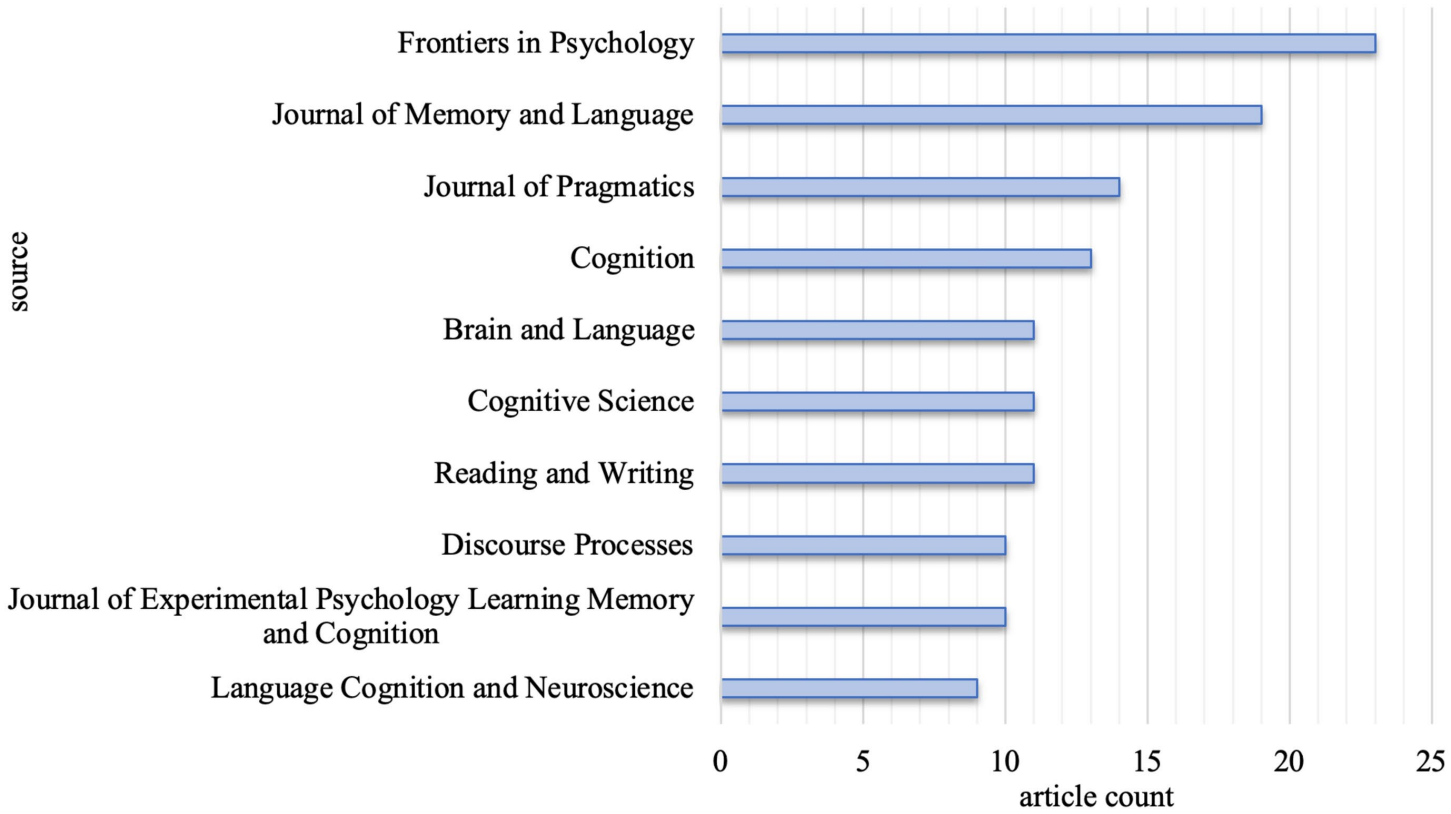
Top ten most productive journals.

Altogether, 472 articles were distributed in 200 journals, 26 of which were identified to have published a minimum of five articles, revealing different research dimensions related to lexical inferencing. Figure 2 shows the top 10 representative journals. Among the journals, the *Frontiers in Psychology* (*n* = 23) published the most articles on lexical inferencing, followed by *Journal of Memory and Language* (*n* = 19) and *Journal of Pragmatics* (*n* = 14).

### 4.2. Document co-citation analysis

**Figure 3:**
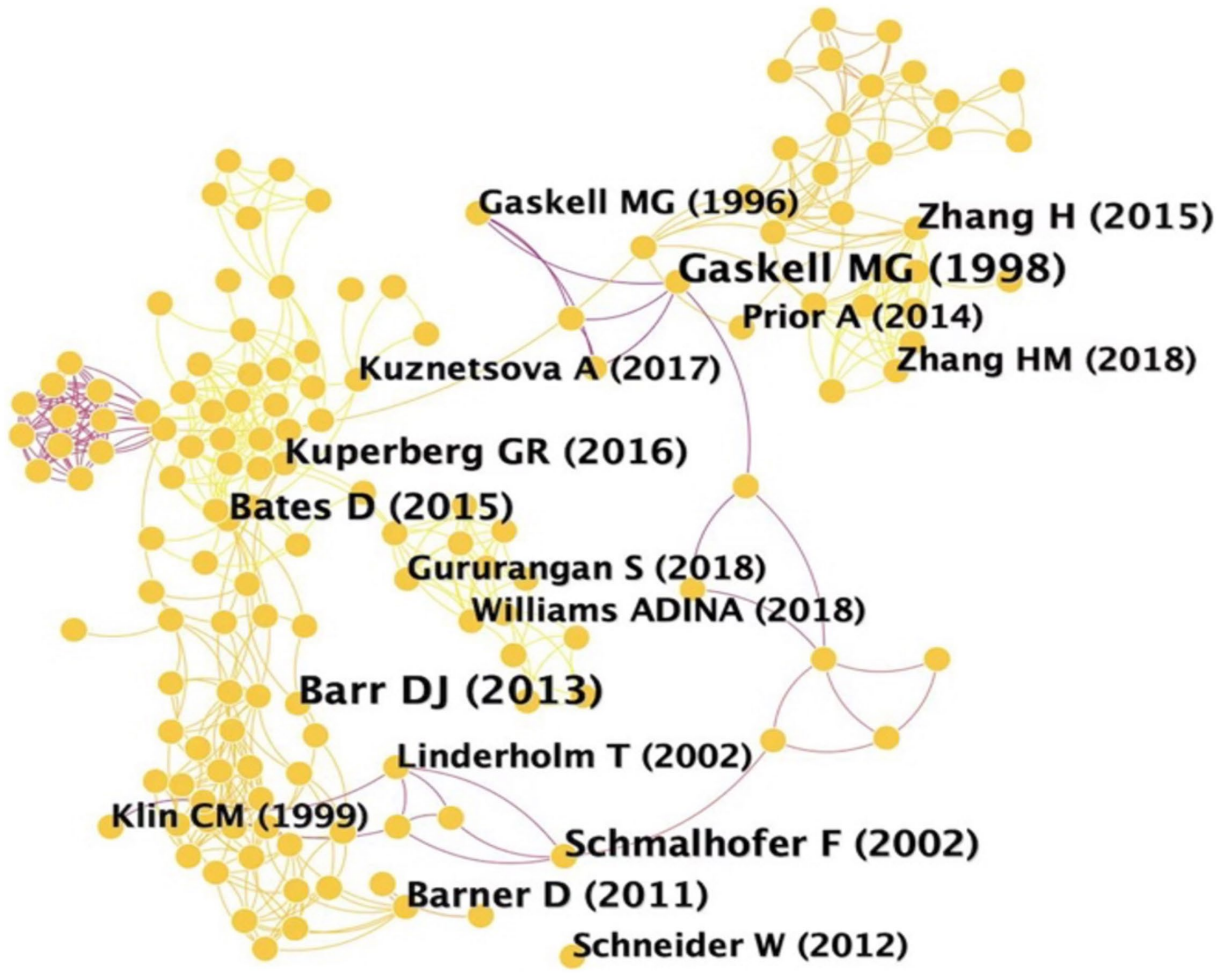
Network map of the most cited articles of lexical inferencing.

A visualized co-citation map was generated based on 472 bibliographic recordings from 2001 to 2021. The threshold was set to top fifty levels of most cited or occurred articles with 1-year time slice. Figure 3 illustrates the top sixteen most cited articles. The network map of co-cited articles produced 682 individual nodes and 1,390 links, demonstrating the number of cited articles and co-citation relationships among the bibliographic datasets, respectively. Table 1 shows the top 7 most cited articles according to the citation counts in the field of lexical inferencing. The first most cited article was written by Gaskell and Marslen-Wilson (1998). Two second most cited articles were written by Zhang (2015) and Zhang and Koda (2018), respectively. Four papers including Barner et al. (2011), Gaskell and Marslen-Wilson (1996), Ke and Koda (2017), and Klin et al.’s (1999b) rank fourth in the top most cited articles.

**Table 1:** The top seven most cited articles in lexical inferencing.

Citation counts	Author (year)	Title	Journal
9	Gaskell and Marslen-Wilson (1998)	Mechanisms of phonological inference in speech perception.	Journal of Experimental Psychology: Human Perception and Performance
7	Zhang and Koda (2018)	Vocabulary knowledge and morphological awareness in Chinese as a heritage language (CHL) reading comprehension ability.	Reading and Writing
7	Zhang (2015)	Morphological awareness in vocabulary acquisition among Chinese-speaking children: Testing partial mediation *via* lexical inference ability.	Reading Research Quarterly
6	Gaskell and Marslen-Wilson (1996)	Phonological variation and inference in lexical access.	Journal of Experimental Psychology: Human Perception and Performance
6	Barner et al. (2011)	Accessing the unsaid: The role of scalar alternatives in children’s pragmatic inference.	Cognition
6	Ke and Koda (2017)	Contributions of morphological awareness to adult L2 Chinese word meaning inferencing.	The Modern Language Journal
6	Klin et al. 1999b)	Forward inferences: From activation to long-term memory.	Discourse Processes

The first and the fourth most cited articles were written by Gaskell and Marslen-Wilson (1996, 1998), respectively. The two studies investigated the mechanism of mapping processing from speech input to lexical representation of phonological form through different cross-modal priming experiments designed to reveal phonological effects on lexical access. Gaskell and Marslen-Wilson (1996) focused on matching process between perceptual input and lexical representations by manipulating two variables, i.e., phonological changed/unchanged and viable/unviable contexts in the experiments. Participants aged 18 to 45 completed the tasks in the pretest, Experiments 1 and 2. The results of ANOVA showed that the effects of phonological variation on lexical access were confined to the segmental contexts, that is, contextual viable/unviable, and indicated that lexical abstract representations were constructed in a context-sensitive processing environment. Gaskell and Marslen-Wilson (1998) extended their study (Gaskell and Marslen-Wilson, 1996) by focusing on the responses to the effects of phonological viability in words and non-words processing. In addition, the study investigated listeners’ perceptual judgments on the form of phonologically variant speech by manipulating three binary variables, phonological change, lexical status, and viability in two phoneme monitoring experiments. Altogether, 149 participants aged 18–45 were recruited in the experiments. The results of ANOVA and ANCOVA revealed the effects of viability of the phonological context on the perception of both words and non-words and that the mapping from surface to underlying forms of speech was the same for words and non-words. The two studies attempted to explore the matching process between the speech input and lexical representation across word boundaries, through which access to semantics is gained. Unlike previous findings that minimal distortion of the tokens of speech was found to disrupt lexical access for isolated words, the results of the two studies demonstrated whether the same deviations cause mismatch or are processed fluently depended on their following segmental context. In addition, the mapping process involved the retrieval of both semantic and phonological knowledge for real words, whereas for non-words, only phonological knowledge was retrieved. This finding indicated that the inferencing in mapping process was not entirely based on access to stored knowledge relating to the meaning of a word.

Two second most cited articles are Zhang (2015) and Zhang and Koda (2018), in which they investigated the interplay between lexical inferencing, morphological awareness, and vocabulary knowledge in contributing to reading comprehension. Zhang (2015) explored the mediating effect of lexical inferencing and Chinese-specific morphological awareness, comprising of derivational, compound, and compound structure awareness, on reading vocabulary knowledge. Two hundred eighty-eight second-grade Chinese-speaking elementary students with an average age of 7.8 years participated in a series of experiments. Hierarchical multiple regressions were conducted to investigate whether morphological awareness and lexical inference ability facilitated reading vocabulary. The results showed that sublexical morphological information, specifically derivational and compound awareness yielded a pivotal contribution to the development of reading vocabulary knowledge, and that lexical inferencing played a mediational role in connecting another facet of morphological awareness, that is, compound structure, with reading vocabulary knowledge. The findings constructed a path model between morphological awareness and vocabulary knowledge and indicated lexical inferencing mediated the indirect relationship between morphological awareness and reading vocabulary acquisition.

Zhang and Koda (2018) identified a possible casual sequence of morphological awareness, vocabulary knowledge, and reading comprehension among 195 students (mean = 20.13 years) of Chinese as a heritage language. Chinese orthography is morphosyllabic and emphasizes more on the connection between the grapheme and the morpheme (Koda, 2007). A morpheme is considered as the minimal linguistic unit that integrates a meaning or grammatical function with a sound (Finegan, 2014). In Chinese, compounds have been widely acknowledged as the most prominent morphological phenomenon, which accounts for about 95% in word formation (Ceccagno and Basciano, 2007). Chinese morphological processing highly involves the structural and functional understandings (Zhang and Koda, 2018). Thus, two factors of morphological awareness (structural and functional awareness), two aspects of vocabulary knowledge (early and textbook/academic vocabulary knowledge), higher-level reading ability (lexical inference ability), and reading comprehension performance were measured through structural equation modeling with a bootstrap estimation method and multiple regression analyses. The research demonstrated vocabulary knowledge and morphological awareness jointly contributed to the participants’ reading comprehension performance. In addition, vocabulary knowledge contributed both directly and indirectly to reading comprehension *via* morphological awareness. Further, academic vocabulary learning was verified to have a significant effect on predicting higher-level reading comprehension ability, whereas early exposure did not facilitate lexical inference ability and reading comprehension process. The results provided new understanding on the relationship between morphological awareness, vocabulary knowledge, lexical inference, ability and reading comprehension ability.

Ke and Koda (2017), ranking fourth in the top most cited articles as well, examined how morphological awareness of L1 and L2 language affected L2 word meaning inferencing among 50 English-speaking learners of Chinese with the average age of 20.3 years. According to Miller (2019), English orthography is one of the deepest alphabetic orthographies, and is highly irregular and inconsistent between graphemes and phoneme; for instance, a single letter could represent multiple sounds and single sounds could be mapped onto multiple letters. English orthography is morphophonemic and word-based in written texts, whereas Chinese orthography is morphosyllabic and character-based in written texts. Thus, Ke and Koda (2017) attempted to investigate the readers’ sensitivity to the grapheme-morpheme relationships through a repeated measures analysis of covariance and two rounds of hierarchical regression analyses. The findings showed L2 adult learners were sensitive to intraword morphologically complex structure and only L1 morphological awareness was identified to transfer and facilitate the development of L2 morphological awareness. In addition, there was no joint contributions of L1 and L2 morphological awareness to L2 word meaning inferencing. Without L2 linguistic knowledge presenting, L2 morphological awareness was observed to affect L2 word meaning inferencing significantly. The results suggested that L2 Chinese learners, even exposing to Chinese-as-a-foreign-language environment at college-level for a few years, were able to grow intraword morphological sensitivity and that there was a significant intralingual effect but no interlingual effect on L2 word meaning inferencing.

Klin et al. (1999b) also ranks fourth. They investigated how forward inferences were activated, processed in working memory, and stored in long-term memory during text reading among 152 undergraduates. Previous studies showed inconsistent findings about the forward inference, which was, as Klin et al. (1999b) claimed, due to methodological issues involved in designing the experiments. They conducted four experiments to examine the influence of text variables on inference process to demystify the discrepant findings. Experiment 1 was designed to examine the effect of text variables which include high- and low-predictable constraints on the activation of forward inferences among readers. The results showed that participants’ naming times of a probe word between two types of texts were insignificantly different. In order to identify the retention of the inferred information in working memory, a target line contradicting the inference was added to high-predictable context in Experiment 2 and low-predictable context in Experiment 3, respectively. The findings in Experiments 2 and 3 revealed that readers retained the inferred information of the unfamiliar words in their working memory while reading. Experiment 4 was to test whether the inference was encoded into the long-term memory using a delayed recall task with different idea units grouped into three categories. The results indicated that the inference was encoded into the long-term memory. The authors then concluded that the forward inferences were activated momentarily, maintained in working memory, and encoded into long-term memory during the reading process. This study more critically stressed the importance of text variables in research on the forward inferences in reading.

Barner et al. (2011) is the fourth most cited article as well. The study investigated the complex process of lexical inferencing among children, specifically quantifier inferencing from the pragmatic perspective. It is known to be especially difficult for children to infer the core meaning of the lexical items and to derive a scalar implicature due to the fact that children have to ascertain the speakers’ implicit meanings which goes beyond the literal meanings. Scalar implicatures are inferences that are based on the use of an informationally weak expression instead of a strong alternative. Barner et al. (2011) examined interpretations of sentences containing *some* and context-specified alternatives among 4-year-old native speakers of English. The findings of the experiments confirmed the hypothesis that children’s insufficient knowledge of scalar alternatives imposed a significant constraint on their computing scalar implicature. Prior to this study, various factors, including limited working memory capacity, lack of understanding of context and meta-linguistic tasks, and processing limits, were also captured to find possible explanations to the failure of children’s quantifier inferencing.

### 4.3. Cluster interpretations

**Figure 4:**
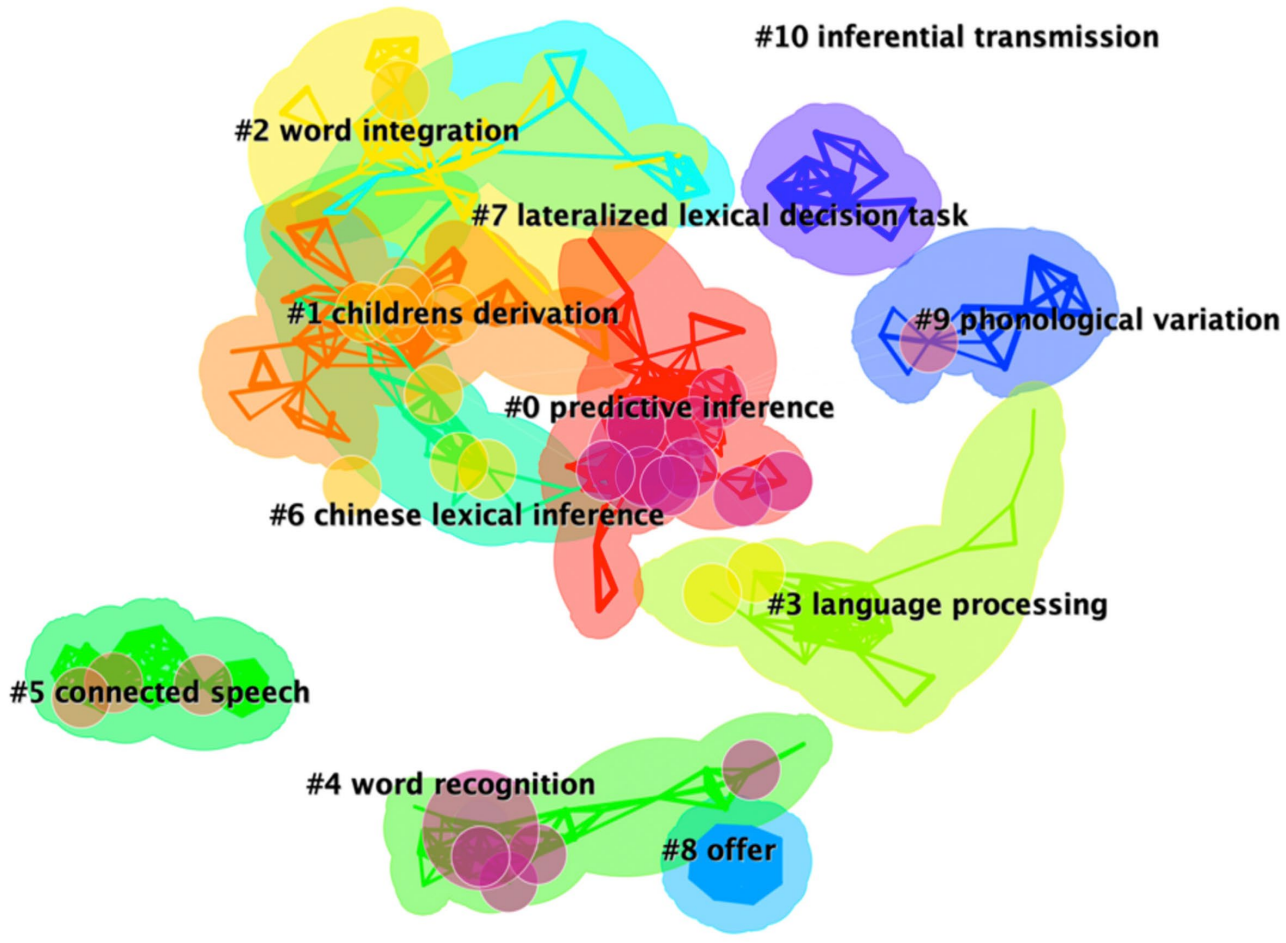
Network map of major clusters of lexical inferencing between 2001 and 2021.

The synthesized network can be divided into co-citation clusters of references, demonstrating intellectual base of a specialty (Chen, 2017). A total of 472 articles generated co-citation maps of the lexical inferencing with 207 clusters, revealing interrelationships and interconnectivity of highly internally homogenous and externally heterogeneous groups. The network has a modularity of 0.94 and the average silhouette score is 0.89. According to Chen (2004), the modularity and the silhouette values are two important metrics to show the overall structural properties of the network of a research domain. The modularity measures the extent to which a network can be decomposed to various modules. The modularity values close to “1” indicate the network of a research domain is clearly divided into distinct modules (Chen et al., 2010; Chen, 2016). The silhouette scores measure the quality of a clustering configuration. The silhouette values close to “1” indicate the contents of references in a cluster are highly consistent (Chen et al., 2010; Chen, 2016). Figure 4 presents the top 11 clusters with labels. Among the top cited clusters, cluster #4, cluster #6, and cluster #1 encompass the articles with the highest citation bursts, revealing the increase of interests in lexical inferencing in the period of 2001–2021. Table 2 shows the major seven clusters with labels and the number of included articles.

**Table 2:** Summary of clusters #0 to cluster #6.

Cluster rank	Terms (LLR)	The number of articles
0	Predictive inference	47
1	Children’s derivation	47
2	Word integration	32
3	Language processing	25
4	Word recognition	24
5	Connected speech	21
6	Chinese lexical inference	18

Cluster #0 is the largest cluster labeled as predictive inferences, which situates research on the conditions leading to the activation of a predictive inference and on the extent to which the predictive inferences are activated and encoded during reading. The cluster has 47 articles and a silhouette value of 0.989. The top three most cited articles are Klin et al. (1999a,b) and Schmalhofer et al. (2002). Klin et al. (1999b) was also the top cited article, whose work focused on the role of text variables in forward inference generation during reading process. Klin et al. (1999a) examined the influence of text variables on predictive inferences during reading and identified the paths of when and how forward inferences were drawn in working memory and encoded into long-term memory. The results showed forward inferences were likely to be activated momentarily if they were highly predictable, which confirmed the minimalist hypothesis and the constructionist theory. The minimalist hypothesis claims inferences are encoded automatically when information is easily accessible from explicit statements in the text or general knowledge during reading (McKoon and Ratcliff, 1992). The constructionist theory proposed by Graesser et al. (1994) argues the knowledge-based inferences are generated on-line when readers comprehend narrative text, provided that certain conditions are satisfied such as when the reader has a specific goal to generate these inferences and the inferences are highly predictable. However, the finding that forward inferences were encoded into long-term memory was inconsistent with that of the previous ones (e.g., Keefe and McDaniel, 1993; Fincher-Kiefer, 1996). The discrepancies could be due to the task difference. Schmalhofer et al. (2002) investigated interconnectivity of predictive and bridging inferences and the results were explained within the framework of the construction-integration theory (Kintsch, 1988, 1998; Singer and Kintsch, 2001). Though predictive and forward inferences were considered as synonyms in the literature, Schmalhofer et al. (2002) distinguished these two types of inferences. They claimed predictive inferences with the explicit focus are about the state of affairs described by the text while forward inferences with the implicit focus are about what an author will say next in the text. Similarly, they distinguished between backward and bridging inferences. Singer (1996) identified backward inferences as a process required a reader to work backward to previously described information when reading the second sentence, and claimed backward inferences undergone a knowledge-independent generation process. Schmalhofer et al. (2002), however, suggested backward inferences were the knowledge-based inference generation process. In addition, they proposed bridging inferences occur when a reader starts reading the first sentence and the elaborations which based on the presented information are later bridged to other domain knowledge that is generated from a subsequent sentence. The construction-integration theory argues that the two types of processing phases, the construction and integration phases, proceed successively during reading comprehension process. By manipulating text variations in terms of computer simulations, Schmalhofer et al. (2002) developed a unified model which consists of three levels of representation, i.e., surface, propositional, and situational representations, which claimed that forward and backward causal inferences exist at the propositional representation while predictive and bridging inferences are grounded at the situational representation. In addition, explicit statements may occur across three representations. Their findings provided insights into the role of construction-integration theory in explaining various results about the generation and persistence of inferences during reading comprehension.

Cluster #1 is the second largest cluster labeled as children’s derivation. Forty-seven articles are included in this cluster with the silhouette value of 0.949. This cluster is mainly presented by Barner et al. (2011), Bott et al. (2012), and Stiller et al. (2015), which explores children’s ability to compute scalar implicatures of inferences in pragmatic context. Barner et al. (2011), as reviewed earlier (see section 3.2.2), investigated children’s ability to compute scalar alternatives in pragmatic context and confirmed the prior hypothesis that children’s ability to make scalar implicatures of lexical items was determined by their knowledge of scalar alternatives. Following the paradigm of Bott and Noveck’s (2004) study, Bott et al. (2012) examined scalar implicatures of inferences that precluded trade-offs between response time and accuracy among university students. Employing a hierarchical model fitting analysis to compare the processing time and accuracy of the sentences with and without implicatures, they found that speed-accuracy strategies may not be the only possible cause to delayed upper-bound interpretations. A distinction was also made between sentence complexity costs including such differences as the memory search and costs pertaining to the inferential process of deriving the implicature. The results were contrary to several different types of processing models, including the default implicature model (Levinson, 2000; Chierchia, 2004) and the contextual model (Sperber and Wilson, 1995). The default model claims the implicature arises automatically and the lower-bound interpretation requires more processing time. The contextual model argues the implicature depends on the contextual situation. The results indicated implicature costs may due to inference mechanism *per se*. In addition, Stiller et al. (2015) assessed preschool children’s (average age of three groups: 2.6, 3.5, and 4.5 years) ability to make *ad-hoc* implicature of pragmatic reasoning, another difficult type of inferences which is constrained by special features of the contexts rather than ordering relations of scalar implicatures. Through logistic mixed-effects models the study reported that preschoolers developed the ability to make contextually grounded inferences, that is *ad-hoc* implicatures. The evidence enabled Stiller et al. (2015) to make a claim that children’s difficulty in computing scalar alternatives of lexical items may be due to their lexical comprehension ability rather than their underlying pragmatic competence.

Labeled as word integration, Cluster #2 has 32 articles and a silhouette value of 0.987. The cluster focuses on clarifying the influences of vocabulary knowledge, inference making and verbal working memory on word-to-text integration in written and spoken language comprehension. Daugaard et al. (2017) examined the effects of vocabulary knowledge and inference making ability on 6th graders’ reading comprehension through an oral vocabulary test and a comprehension test by taking verbal working memory into account as an explanatory variable. The results replicated the finding that inference making facilitated the process of selecting the precise word meanings in texts (Cromley and Azevedo, 2007; Ahmed et al., 2016; Segers and Verhoeven, 2016). The vocabulary knowledge, however, was not considered as a significant mediator in inference making process during reading, which provided evidence against an alternative mediation hypothesis that suggests vocabulary mediates the effect of the inference making during language comprehension. Although there was a correlation among inference making ability, vocabulary knowledge and verbal working memory, no significant difference was found between vocabulary knowledge and verbal working memory. The findings indicated the retrieval of the semantic word knowledge was not mediated by verbal working memory. The study also observed that the process of reading comprehension was considered to involve both lower-level skills (the retrieval of lexical knowledge and extract of word meanings) and advanced skills (inference making skills).

Cluster #3 is labeled as language processing and has 25 articles with a silhouette value of 0.989. Kuperberg and Jaeger (2016) reviewed the studies pertaining to language processing and argued that the constructs with regard to probabilistic prediction in language processing interacted within a multi-representational hierarchical framework which has been proposed to explain the complex cognitive processing since the comprehender has to draw upon multiple different types of stored information. The framework considers message-level representations as probabilistically generating information at these multiple levels of representation. Other studies on language processing mainly investigated neurocognitive processing of prediction at multilevel representations in language comprehension with ERP and eye-tracking experiments (Ryskin et al., 2019; Kuperberg et al., 2020; Leckey and Federmeier, 2020; Feng et al., 2021). Employing sentence reading tasks, Kuperberg et al. (2020) examined whether the brain engaged different neurocognitive mechanisms in response to words (critical nouns in three-sentences scenarios) that confirmed and/or violated strong predictions at *High constraint* and *Low constraint* contexts during language comprehension among 39 native English speakers (mean = 21.6 years). By examining different ERP components such as N400, late frontal positivity, and the posterior positivity/P600 through a series of linear mixed-effect regression models, the study found solid evidence that fulfilling and violating predictions at different levels of representation involved distinct temporal and spatial segregation of neural activity. Employing eye-movement technique, Ryskin et al. (2019) investigated the modulation of lexical inferences under pragmatic contexts among 238 university students who were fluent speakers of English. By adopting a multilevel linear regression and autoregressive logic mixed-effect models, Ryskin et al. claimed that listeners retrieved numerous sources of information to achieve pragmatic communication.

Cluster #4 labeled as word recognition includes articles from Gaskell and Marslen-Wilson (1996, 1997, 1998) and Connine et al. (1997) with the major interest in the interconnectivity of phonology, lexicon and semantics in spoken word recognition. The cluster has 24 articles and a silhouette value of 1. Connine et al. (1997) investigated the underlying process of spoken word recognition through four experiments. A total of 284 participants were recruited in the study and the data were analyzed by ANOVA. The results showed that the degree of similarity between phonetic input and a stored lexical representation consisting of both form and meaning determined the activation of lexical representation. Gaskell and Marslen-Wilson’s (1996, 1998) focused on the lexical inference with regard to viability of phonological contexts from the perspective of the TRACE model (McClelland and Elman, 1986) and the connectionist model (Gaskell, 1994; Gaskell et al., 1995). The TRACE model of speech perception claims that a very large number of simple processing units undergo an activation and competition through the excitatory and inhibitory interaction. However, this model might show the influence of viability of phonological context at the phoneme level rather than at the word level (i.e., across word boundaries). The connectionist model uses a recurrent network which is trained to map from surface (speech input) to the underlying representations. Gaskell and Marslen-Wilson (1997) elaborated on the distributed feature of connectionist theory which argued the retrieval of lexical representations in word recognition involved a distributed pattern encompassing the combination of lexical variables with abstract phonological information.

Other meaningful clusters, such as cluster #5 (connected speech) and cluster #6 (Chinese lexical inference), are also worth mentioning. Cluster #5 mainly focuses on how phonological variations with systematic changes or other factors, such as orthography, and frequency effect, activated lexical representation in spoken word recognition. Gow and Im (2004) examined the role of language-specific mechanism and universal perceptual mechanism in phonological processes of assimilation context effects through ANOVA. Two hundred and forty-eight adults between the age of 18–53 served as participants in the four experiments. The findings demonstrated that context effects depended on universal perceptual mechanisms which intertwine with the acoustic properties of phonological modification. Ranbom and Connine (2007) investigated five hypotheses that account for recognition of phonological variation in spoken language comprehension, i.e., underspecification representation, inferential process, feature parsing, tolerance of mismatch, and frequency-based representation among 199 undergraduates. The data were analyzed by ANOVA and the findings were in line with the assumption that strength of activating lexical representation is influenced by production frequency. The study identified that lexical representations were formed by a general model that regulated language organization and process. Recent studies in cluster #6 labeled as Chinese lexical inference center on the roles of lexical inference, vocabulary knowledge, and morphological awareness in reading comprehension performance among monolingual speakers, L2 learners and learners of Chinese as a heritage language. The related studies observed different contributions of lexical inferencing, vocabulary knowledge, and morphological awareness to reading comprehension (Prior et al., 2014; Zhang, 2015; Ke and Koda, 2017; Zhang and Koda, 2018). Prior et al. (2014) examined whether language proficiency and reading abilities played predictive roles in explaining individual differences in L2 lexical inferencing under the framework of the “simple view of reading” model (SVR; Gough and Tunmer, 1986; Hoover and Gough, 1990). The SVR model posits that both decoding and linguistic comprehension are necessary for skilled reading. Fifty-three 12th graders with Russian as their L1 and Hebrew as an L2 were recruited in the study. The results of two multiple regression analyses showed L2 vocabulary knowledge and single word decoding accuracy contributed to L2 reading comprehension, which in turn was closely related to learners’ lexical inferencing ability in their L2.

The top major clusters revealed a diverse range of research in lexical inferencing. Lexical inferencing was discussed under the phonological models, such as the TRACE model (McClelland and Elman 1986) and the connectionist model (Gaskell, 1994; Gaskell et al., 1995), and under the pragmatic models, such as the default implicature model (Levinson, 2000; Chierchia, 2004) and the contextual model (Sperber and Wilson, 1995). The minimalist hypothesis (McKoon and Ratcliff, 1992) and the constructionist theory (Graesser et al., 1994) helped to demystify the generation process of lexical inferencing with regard to when and how inferences were drawn in working memory and encoded into long-term memory. More recent studies centered on the interconnected relationship among lexical inferencing, vocabulary knowledge, morphological awareness, and reading comprehension through path analyses and mediation analyses. Other recent studies attempted to explore how lexical inferencing works by using eye-tracking methods, ERP, etc. from psychological and neurological aspects.

### 4.4. Hot topics for lexical inferencing

**Figure 5:**
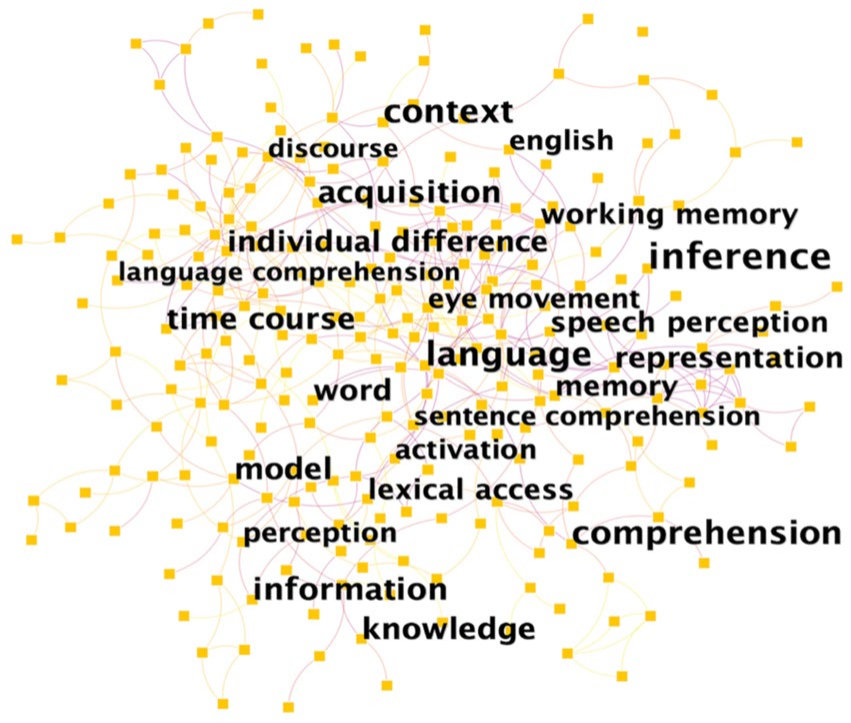
Top 23 most frequent co-occurring keywords in lexical inferencing research.

Hot topics can be featured by a list of most important keywords and the analysis of keywords identifies the intellectual structure of a specialty (Chen, 2012). Figure 5 illustrates the top 23 keywords in lexical inferencing research. For the analysis, the top ten most frequent keywords which include inference, context, language, comprehension, information, acquisition, knowledge, time course, model, and word were discussed. Table 3 shows the top ten keywords of lexical inferencing research along with their frequency counts.

**Table 3:** The top ten most frequent co-occurring keywords in lexical inferencing.

Rank	Keywords	Counts
1	Inference	92
2	Context	55
3	Language	55
4	Comprehension	54
5	Information	43
6	Acquisition	43
7	Knowledge	34
8	Time course	33
9	Model	32
10	Word	30

The keyword co-occurrence analysis in lexical inferencing reveals several active research topics such as inferencing based on various information (e.g., contextual effect), language acquisition and comprehension, and time course as an indicator of activation and retention of lexical inferencing.

One of the top keywords is inference which is a distinct topic that has received consideration in the domain of lexical inferencing. Inference, according to Keenan et al. (1990), is widely used in research as a synonym of inferencing. The research on lexical inferencing emphasized the role of inferencing in decoding and encoding the unfamiliar words during language comprehension. Various information, such as contextual effect and linguistic information background, may affect the process of lexical inferencing in language comprehension. Identifying appropriate meanings of unfamiliar words involves making use of these information. A growing body of research has specifically investigated the extent to which lexical inferencing was intertwined with the linguistic information such as vocabulary knowledge and morphological awareness in language comprehension. Zhang and Koda (2012) found that morphological awareness had a direct effect on vocabulary knowledge development and an indirect contribution to vocabulary knowledge development *via* lexical inferencing ability. However, none of the three variables, i.e., vocabulary knowledge, morphological awareness, and lexical inferencing ability were found to have any significant independent contribution to reading comprehension when the other two variables were controlled for. Other studies, on the contrary, have provided evidence for the contribution of morphological awareness to reading comprehension (Park, 2004; Zhang and Koda, 2018).

Contextual effect was one of the most influential factors in identifying relatedness between morphological awareness, vocabulary knowledge, and lexical inferencing in reading comprehension performance. Contextual cues could be employed by readers or listeners or communicators to comprehend and predict the word meanings. For instance, the findings of the research on lexical inferencing at the phonological-lexical interface demonstrated inconsistent predictions in lexical access (Cook et al., 2001; Schmalhofer et al., 2002; Gow and Im, 2004; Sumner and Samuel, 2005). The controversy may be attributed to different phonetic contexts or sentence contexts or situational-based contexts (e.g., strong and weak context to activating lexical access), which indicated that lexical inferencing in phonological-lexical interface was highly constrained by contextual environment. Another scenario was that contextual variations influenced the role of lexical inferencing during reading. Data from several studies suggested that the predictivity of lexical inference was strongly affected by the length of reading materials as well as the relevance between target and added reading materials (Klin et al., 1999a,b).

The role of lexical inferencing has been widely investigated in the domain of language comprehension and acquisition. Lexical inferencing ability determines the quality of written and spoken language comprehension. Learners will not be able to understand a text if they failed to decode a reasonable number of the words (Oakhill et al., 2019). In addition, lexical inferencing also plays a crucial role in L1 and L2 acquisition. L1 readers tend to infer the meaning of the unfamiliar words based on the background knowledge and the contextual information. Learners’ L1 knowledge has also been documented to contribute to the L2 text comprehension, particularly mediated by lexical inferencing (e.g., Ke and Koda, 2017; Zhang et al., 2019). Koda and Miller (2018) also reported learners’ L1 morphological awareness could be a shareable resource for L2 word meaning inference. Some researchers constructed path models to identify the path routes between morphological awareness, lexical inference and reading comprehension (Zhang et al., 2021).

The time course of lexical inferencing involves the processes of activating, encoding, and retrieving lexical information over time in readers’ memory. It serves as one of the indicators of measuring the effect of lexical inferencing on language comprehension since it reflects readers’ psychological and cognitive process. Some researchers attempted to address the question of the likelihood of the activation of inferencing and its retention in memory by manipulating task variables (Klin et al., 1999a,b; Linderholm, 2002). These studies determined when and how inferencing in reading was activated and maintained in working memory and stored in long-term memory in different tasks according to reading times of the passages and reaction times to the target words. Currie and Cain (2015) also investigated the role of working memory and linguistic information such as vocabulary knowledge in reading comprehension. They reported that vocabulary knowledge directly facilitated inference making in discourse comprehension whereas the contribution of working memory to discourse comprehension was mediated by vocabulary knowledge.

The above-mentioned keywords were hot topics that were constantly discussed in lexical inferencing domain over the past two decades, which captured the dynamics of underlying trend in the knowledge domain of lexical inferencing. Information that can affect lexical inferencing process and the role of lexical inferencing in language comprehension have received considerable critical attention. In addition, most studies focused particularly on the time course of lexical inferencing in language acquisition and comprehension. Further, as can be seen in Figure 5, topics such as individual difference, eye movement, speech perception, lexical access may also reveal the evolving profile of lexical inferencing.

## 5. Discussion

The present study visualized document co-citation analysis of 472 articles pertaining to lexical inferencing between 2001 and 2021 *via* CiteSpace software. The findings of the study substantiated by the critical articles, intellectual base, and hot topics provided a reliable historiography and an overview of knowledge mapping of research on lexical inferencing.

The results of document co-citation analysis revealed a number of prominent clusters which include the probability of activating and retaining inferencing in working memory and storing in long-term memory, children’s derivation in lexical inferencing in pragmatic context, word integration and recognition in reading comprehension and connected speech, psychological and neurocognitive processes underlying language processing mechanism, and the influence of lexical inferencing on reading among learners with Chinese as their L1 and L2. These major clusters represented a diverse range of research domain in lexical inferencing. Hot topics focused on the information that affected lexical inferencing in language acquisition and comprehension as well as the time course that related to the process of inferencing in learner’s memory. Prior research on lexical inferencing observed inconsistent findings with regard to when and how lexical inferencing was activated, retained, and encoded in memory. In addition, lexical inferencing played either direct or indirect role in written and spoken language comprehension. The discrepancies may be attributed to learner-related and discourse-related differences.

Learners’ differences in inferential processing are closely related to their language level, morphological awareness, L1 and L2 learning background, vocabulary knowledge, as well as working memory. Some studies observed the role of morphological awareness in reading comprehension was mediated by lexical inferencing ability (Zhang and Koda, 2012), while other studies found morphological awareness directly facilitated reading comprehension (Park, 2004; Zhang and Koda, 2018). The inconsistent findings may be attributed to participants’ differences. The participants recruited in these studies included preschoolers, primary school students, undergraduate and graduate learners, and learners with different heritage language backgrounds. In addition, readers’ breadth and depth of vocabulary knowledge has been found to be closely related to their lexical inferencing performance. Vocabulary knowledge highly predicted the lexical inferencing ability in reading comprehension. Several studies have indicated that high vocabulary knowledge, in particular the depth of vocabulary knowledge, significantly enhanced readers’ ability to infer unfamiliar words in the text (Qian, 2004; Nassaji, 2006; Marzban and Hadipour, 2012). Working memory, the memory system of storing and processing information simultaneously (Baddeley and Hitch, 1974), is another influential factor in lexical inferencing since readers and listeners need to associate the activated memory of previously processed information with the information being processed currently. Working memory capacity was one of the predictors of drawing lexical inferencing during reading comprehension. High working memory capacity reduced the time course of lexical inferencing (Estevez and Calvo, 2000). Meanwhile, prior research showed that the effect of working memory was also mediated by learners’ vocabulary knowledge on inference making (Chrysochoou et al., 2011; Currie and Cain, 2015). The vocabulary knowledge stored in the long-term memory may also help to retrieve more accurate and available representations for working memory (Nation et al., 1999; Walker and Hulme, 1999).

Additionally, discourse-related differences affect the role of lexical inferencing in language comprehension. Variables, such as length and place of added paragraphs to the target passage, and the relevance of added lines to the target words, created different environments for drawing, encoding and maintaining lexical inferencing in readers’ memory during reading. Response times were measured to show the extent to which the length and place of additional materials in controlled contexts determined the inferencing process. Linderholm (2002) observed the working memory capacity and the causal text constraints were of importance in inferencing during discourse comprehension. Context with sufficient clues is one of the premises for the successful inferring of the word meaning. Learners were more likely to infer the word meaning accurately with both morphological and contextual information available (Zhang et al., 2022). The findings suggested readers’ ability to infer the meanings of novel words was promoted when contextual cues were more explicit. Evidence from phonological-lexical studies also proved the phonological changed/unchanged and viable/unviable contexts existed at multiple levels such as phonetic-level, sentence-level, and situational-level accounted for the contradictory findings with regard to when and how the inferencing was activated. Further, contextual differences worked with word transparency, the degree to which the word is related to the meaning of individual morphemes, to predict the accuracy of lexical inferencing. Chen (2019) investigated how the factors such as L2 learners’ morphological awareness and vocabulary knowledge affected lexical inferencing for opaque, semi-transparent and transparent words during reading. The results revealed different levels of word transparency influenced the role of morphological awareness and vocabulary knowledge in lexical inferencing. Tang and Chan (2022) argued that words with three levels of transparency affected both sentence- and passage-level reading. The interactions between word transparency and context length as well as between word transparency and L1 background were also observed for inferencing accuracy.

## 6. Conclusion and future work

Through bibliometric analysis, the present study not only identified the intellectual base and structure of the scientific domain of lexical inferencing, but also characterized the hot topics of lexical inferencing. A growing number of publications in lexical inferencing can be observed over the past two decades. The top prolific journals in lexical inferencing are *Frontiers in Psychology*, *Journal of Memory and Language*, *Journal of Pragmatics*, *Cognition*, and *Brain and Language*. The results of co-cited references analysis, landscape view of clusters, and keyword co-occurrence analysis demonstrated the research has documented the impacting factors such as learner-related and discourse-related differences of lexical inferencing in language comprehension. Overall, the present study represents the first extensive examination of lexical inferencing and could offer some important insights into the complex nature of lexical inferencing.

Despite the existing results, further studies with more focus on the effects of emotional factors, including learners’ motivation, attitudes on lexical inferencing, should also be taken into consideration. Given the fact that emotional processes involve complicated mechanism of cognitive processes (Kleinginna and Kleinginna, 1981; Seth, 2013), there is abundant room for further progress in identifying the role of emotions in lexical inferencing. In addition, much of the literature has paid particular attention to psychological and cognitive processes using behavioral experiments, while the research on neural correlates of lexical inferencing and language processing remains a major challenge. As suggested by Wang et al. (2018), relatively little research investigating neural patterns in processing inferences was conducted and more research on neural mechanism underlying various types of inferencing is needed. How the brain is activated is a vital attribute to language processing as well, which may contribute greatly to our understanding and interpretation of the intricate relations between the human brain and the language processing mechanism. Further studies would also be worthwhile regarding cross-linguistic studies in lexical inferencing.

## Author contributions

HY contributed to the conception and visualization of the study, and wrote the draft of the manuscript. LF conceived the idea, reviewed the manuscript, and provided the feedback. HSY contributed to writing and editing. All authors contributed to the article and approved the submitted version.

## Funding

This study was supported by the Double First-Class Discipline Major Project of Beijing Foreign Studies University (2022SYLZD008) to LF, and the National Social Science Fund of China (22BYY185) to HSY and HY.

## Conflict of interest

The authors declare that the research was conducted in the absence of any commercial or financial relationships that could be construed as a potential conflict of interest.

## Publisher’s note

All claims expressed in this article are solely those of the authors and do not necessarily represent those of their affiliated organizations, or those of the publisher, the editors and the reviewers. Any product that may be evaluated in this article, or claim that may be made by its manufacturer, is not guaranteed or endorsed by the publisher.
